# Multiplicity of Glycosphingolipid-Enriched Microdomain-Driven Immune Signaling

**DOI:** 10.3390/ijms22179565

**Published:** 2021-09-03

**Authors:** Noriko Yokoyama, Kei Hanafusa, Tomomi Hotta, Eriko Oshima, Kazuhisa Iwabuchi, Hitoshi Nakayama

**Affiliations:** 1 Institute for Environmental and Gender-Specific Medicine, Juntendo University, Graduate School of Medicine, Urayasu, Chiba 279-0021, Japan; n-yokoyama@juntendo.ac.jp (N.Y.); k.hanafusa.nt@juntendo.ac.jp (K.H.); t-hotta@juntendo.ac.jp (T.H.); e.osuka.wc@juntendo.ac.jp (E.O.); iwabuchi@juntendo.ac.jp (K.I.); 2 Laboratory of Biochemistry, Juntendo University Faculty of Health Care and Nursing, Urayasu, Chiba 279-0023, Japan; 3 Infection Control Nursing, Juntendo University Graduate School of Health Care and Nursing, Urayasu, Chiba 279-0023, Japan

**Keywords:** glycosphingolipids, microdomain, immune signaling

## Abstract

Glycosphingolipids (GSLs), together with cholesterol, sphingomyelin (SM), and glycosylphosphatidylinositol (GPI)-anchored and membrane-associated signal transduction molecules, form GSL-enriched microdomains. These specialized microdomains interact in a *cis* manner with various immune receptors, affecting immune receptor-mediated signaling. This, in turn, results in the regulation of a broad range of immunological functions, including phagocytosis, cytokine production, antigen presentation and apoptosis. In addition, GSLs alone can regulate immunological functions by acting as ligands for immune receptors, and exogenous GSLs can alter the organization of microdomains and microdomain-associated signaling. Many pathogens, including viruses, bacteria and fungi, enter host cells by binding to GSL-enriched microdomains. Intracellular pathogens survive inside phagocytes by manipulating intracellular microdomain-driven signaling and/or sphingolipid metabolism pathways. This review describes the mechanisms by which GSL-enriched microdomains regulate immune signaling.

## 1. Introduction

**Figure 1:**
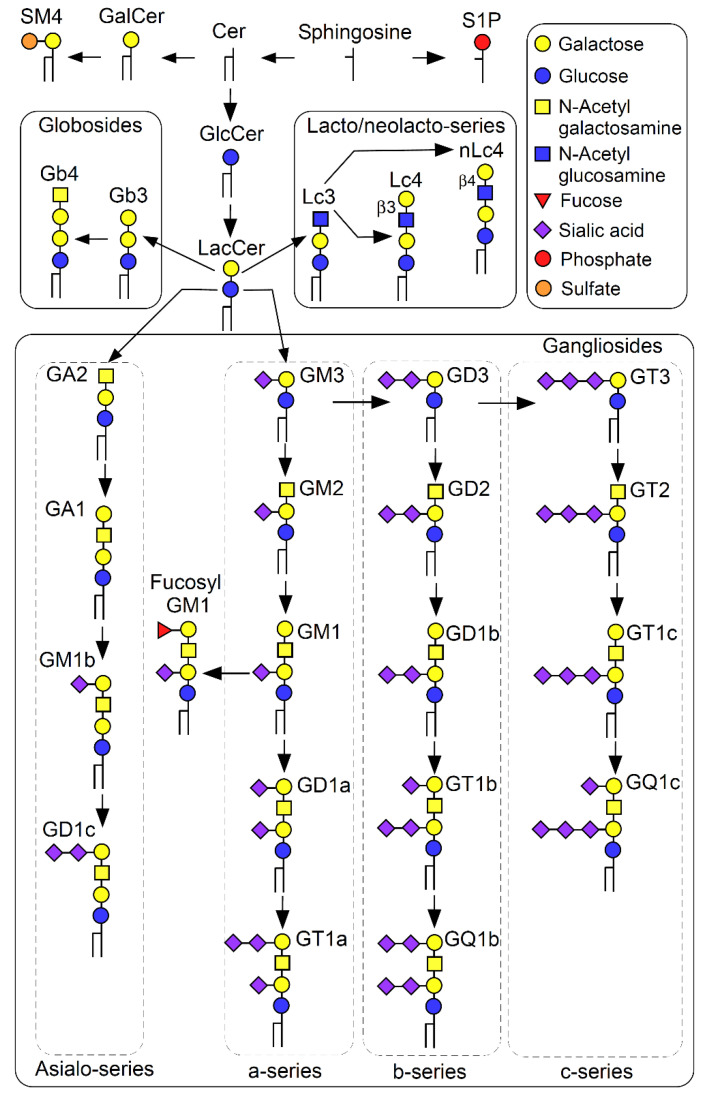
Schematic pathway of GSL biosynthesis. GSLs and the related molecules referred to in this review are shown.

**Figure 2:**
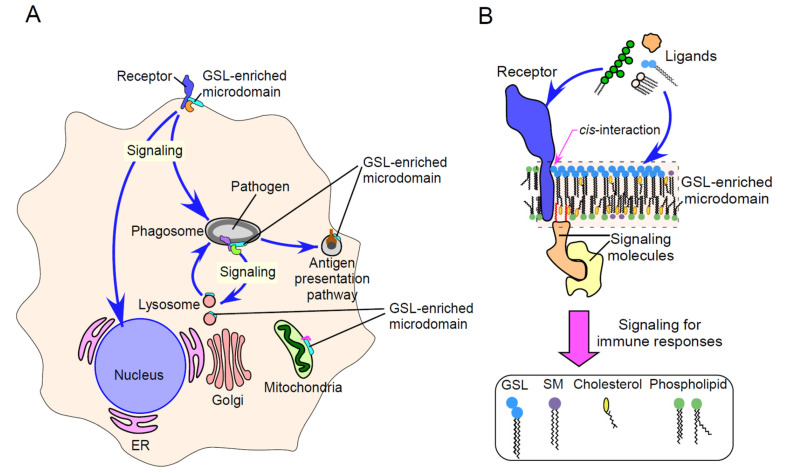
Multiplicity of GSL-enriched microdomain-driven immune signaling. (**A**) Schematic image showing multiplicity of GSL-enriched microdomain-driven signaling in immune cells. In both innate and adaptive immunity, GSL-enriched microdomains affect immune signaling by themselves or by *cis-*interaction with various immune receptors. This results in various immune responses, such as cytokine production, phagocytosis/phagosome maturation, antigen presentation and apoptosis. GSL-enriched microdomains are present not only in plasma membranes but also in membranes of intracellular organelles, such as lysosomes and phagosomes. LacCer forms membrane microdomains on intracellular granules, including lysosomes, in human neutrophils, and these LacCer-enriched microdomains on phagosomes act as a platform of intracellular signaling required for phagosome maturation (fusion of lysosomes to pathogen-containing phagosomes). GD3 forms membrane microdomains on mitochondria-associated membranes (MAMs) and contributes to autophagosome assembly. ER, endoplasmic reticulum. Golgi, Golgi apparatus. (**B**) Schematic image showing *cis*-interactions between GSL-enriched microdomains and immune receptors. The transfer of signals induced by ligand binding into cells involves the direct binding of these ligands to GSL-enriched microdomains in plasma membranes, followed by the transduction of the signals through membrane-associated signal transduction molecules. In addition, signaling molecule-associated GSL-enriched microdomains interact in *cis* with various receptor proteins, leading to a variety of immune responses. Thus, GSL-enriched microdomains provide signaling platforms for ligand binding to the plasma membranes of immune cells. Intracellular GSL-enriched microdomains may provide platforms for cross-talk among several types of proteins, such as membrane-associated and signaling proteins and sphingolipid metabolites.

Membrane microdomains, also called lipid rafts, consist of glycosphingolipids (GSLs), cholesterol, sphingomyelin (SM), glycosylphosphatidylinositol (GPI)-anchored proteins and membrane-associated signal transduction molecules [1,2]. GSLs, a predominant component of microdomains, are characterized structurally by their hydrophobic ceramide and hydrophilic glycan moieties. The ceramide moiety contains fatty acid chains that vary widely in length [3]. More than 400 types of GSLs have been identified based on differences in their glycan structures [4]. Cell surface receptors bind ligands expressed on other cells (in *trans*) to communicate with neighboring cells, whereas a large number of cell surface receptors interact with ligands expressed on the same cell (in *cis*) [5]. The hydrophobic ceramide moiety enables GSLs to interact with the steroid ring system of cholesterol via van der Waals forces and hydrogen bonds [6,7]. In addition, the hydrophilic glycan moieties of GSLs interact in *cis* with each other, promoting the lateral interaction of GSLs with other components of cellular membranes. These interactions result in the phase separation of GSL-enriched membrane microdomains. GSL-enriched microdomains in the outer surfaces of membranes are able to associate with membrane proteins and lipid-anchored signal transduction molecules, which are localized in the inner surfaces of membranes [8,9,10]. These supramolecular complexes provide signaling platforms for cellular functions. The types of GSLs and their metabolism are not only cell type specific but also depend on whether the cells are proliferating or differentiating [4,11]. GSL-enriched microdomains are therefore thought to be involved in a large number of biological functions, including immunological functions (Figure 1 and Figure 2). This review describes the mechanisms by which GSL-enriched microdomains modulate receptor functions and signal transductions in immune signaling, focusing generally on findings in our laboratory.

## 2. Physicochemical Properties of GSL-Enriched Microdomains

A biological membrane consisting of a lipid bilayer is often compared to a sea of phospholipids and cholesterol with floating sphingolipids and membrane proteins. Depending on their physicochemical properties, membrane components are distributed in a non-homogeneous manner throughout the cell membrane, leading to the formation of membrane microdomains that differ in molecular composition. These microdomains form supramolecular structures, which are stabilized by lateral intermolecular interactions. The properties of membrane components provide physical boundaries between the outside and the inside of cells. GSLs are specifically expressed in the outer layer of cell membranes [2]. GSLs can form clusters on cell membranes by lateral interactions based on their physicochemical properties, including hydrogen bonds from hydroxyl groups, the acetamide structure of the ceramide moiety and van der Waals interactions between hydrocarbon chains [11]. The physicochemical properties of GSLs suggest that they form defined clusters and that certain proteins cannot undergo free and continuous lateral diffusion in the membrane but rather are transiently confined to microdomains [4,12,13]. Because there are many difficulties evaluating GSL-enriched membrane microdomains in their original state, the actual state of GSL-enriched membrane microdomains remains unclear. Techniques to determine the structural and molecular arrangements of GSL-enriched membrane microdomains include single-molecule fluorescence tracking and electron microscopy. Although several GSL analogues have been generated by attaching a fluorescent label to the carbohydrate or lipid portion, it is unclear whether these analogues behave identically to natural unlabeled molecules [14]. Recently, hydrophilic fluorophore-conjugated analogues of gangliosides (sialylated GSLs), such as GM3, GM2 and GM1, were shown to be useful for the assessment of these microdomains, because these analogues retain binding specificity to their ligands [15,16]. Fluorescent-labeled SM analogues have been developed, consisting of hydrophilic fluorophores conjugated to the choline headgroup of SM via a hydrophilic nonaethylene glycol linker, which retain their positive charge [17]. These analogues have been shown to behave similarly to native SM in artificial liquid ordered (Lo)–disordered (Ld) phase-separated giant unilamellar vesicles (GUVs) and detergent-resistant membranes (DRMs) from cells.

The sizes of lipid microdomains range from 10 to 200 nm in diameter [1]. In comparison, GSL-enriched microdomains, consisting of GSLs that assemble laterally due to their physicochemical properties, have been reported to be 50–100 nm in diameter [18,19]. Immunoelectron microscopy showed that the neutral GSL lactosylceramide (LacCer, CDw17) forms microdomains with a diameter of about 43 nm in the plasma membranes of resting human neutrophils [18]. The phagocytosis of microorganisms by neutrophils induced the formation of larger LacCer-enriched microdomains, about 60 nm in diameter [20], on phagosomal membranes, suggesting that the reorganized LacCer-enriched microdomains provide platforms for transducing phagosome–lysosome fusion through molecular interactions, such as protein–lipid interactions. Single-molecule imaging with fluorescently labeled GPI-anchored proteins biophysically determined that these microdomains are dynamic domains with a diameter of about 10 nm [21,22]. Thus, the sizes of microdomains depend on the types of observed molecules. These differences may depend not only on the experimental methods used for observation but also on the physical properties of the observed molecules. Unlike GPI-anchored proteins, carbohydrates in GSLs are almost the same as ceramide, and the melting point of neutral GSLs is above 65 °C [23]. Therefore, the lateral interactions among GSLs appear to be very strong, enabling GSLs to pack together in plasma membranes. Acidic GSLs, such as gangliosides, have a much lower melting point than neutral GSLs. Moreover, the phase transition temperature of reconstructed ganglioside-enriched microdomains is around 40 °C, indicating that these microdomains can exist as distinct domains on phospholipid temperatures at body temperature [24]. Indeed, GSLs with different types of glycan structures have been shown to constitute distinct microdomains on the same cells and to have distinct functions [19,25]. Moreover, the interdigitation of long acyl chain SM in the outer leaflet and phosphatidylserine (PS) in the inner leaflet of the plasma membrane has been reported [26,27]. Interestingly, GM1 containing C16:0 but not C16:1 acyl chains was reported to form nanoclusters without the ligand cholera toxin B subunit (CTxB) [28]. Moreover, CTxB was found to induce the co-clustering of GM1 containing C16:0 with the GPI-anchored protein CD59, whereas such co-clustering did not occur with GM1 containing C16:1. These results suggest that the acyl chain structures of the ceramide moiety in GSLs also affect the properties of microdomains.

## 3. GSL-Enriched Microdomains as Regulators of Immune Receptor Signaling

Following the binding of ligands to membrane receptors, the receptors undergo conformational changes, transferring signaling into the cells. During this process, GSL-enriched microdomains interact with various receptor proteins in *cis*, inducing physiological and immune system activities (Figure 1). This section describes the mechanisms by which receptors are regulated by GSL-enriched microdomains. The best known mechanisms are ganglioside–receptor protein interactions. Lateral interactions between GM3-enriched microdomains and insulin receptors (IRs) through a basic lysine residue (K944) lead to the inhibition of insulin-induced signaling in adipocytes [29]. Therefore, an increase in GM3 expression in cells may result in insulin resistance. Moreover, in the absence of epidermal growth factor (EGF), autophosphorylation of the EGF receptor (EGFR) is suppressed by its *cis*-interaction with GM3 through a lysine residue (K642) [30]. Adipogenic stimulation of adipose progenitor cells induces the accumulation of GM3- and caveolin 1-positive microdomains at the base of the primary cilia [31]. This accumulation, however, is reduced by the loss of trichoplein, resulting in the inhibition of IR/insulin-like growth factor-1 (IGF-1) receptor (IGF1R)–Akt signaling and protection from obesity and metabolic syndrome. Molecular dynamics (MD) simulations have shown that GM3 binds to the extracellular domain of the glucagon receptor, a class B1 G-protein-coupled receptor (GPCR), and modulates the dynamics of the extracellular domain, suggesting that GM3 plays a role in regulating the insulin/glucagon signaling ratio [32]. Thus, GM3-enriched microdomains appear to be critical for metabolic regulation. An evaluation of these GM3-receptor interactions suggested that an increase in GM3 content involved the glucose-induced inhibition of IGF1R–Rac1 signaling, affecting keratinocyte motility [33]. This finding suggests possible therapeutic approaches for treating wounds in patients with diabetes.

In addition to GM3, GM1, which is most abundant in neurons, interacts in *cis* with neurotrophin receptors [34] and modulates laminin-1-induced neurite outgrowth via TrkA and β_1_ integrin [35]. A study using tritium-labeled GM1 photoactivable derivatives suggested mechanisms by which GM1 interact in *cis* with receptors, such as TrkA [36]. The formation of the GM1–TrkA complex through those oligosaccharide interactions was found to promote neuroblastoma cell differentiation. In addition, GM1 was found to interact in *cis* with the GPCR serotonin-1A receptor [37]. Most (>90%) gangliosides in adult mammalian brains are composed of GM1, GD1a, GD1b and GT1b, which differ in the number and position of sialic acids linked to a common tetrasaccharide core [38]. GT1b is particularly recognized by botulinum neurotoxin type B (BoNT/B) [39], with a GT1b–synaptotagmin (SYT) *cis*-interactive molecular complex constituting a high-affinity BoNT/B receptor [40]. Similar to the *cis*-interactions of GM3 with IR and EGFR, a lysine residue (K52) on the SYT-juxtamembrane was found to be critical for the SYT–GT1b *cis*-interactions required for BoNT/B binding.

GSL-enriched microdomains are also associated with receptor-mediated immune signaling (Table 1). In innate immune responses, various pattern-recognition receptors (PRRs), including TLRs and integrins, are crucial for the detection of invading pathogens. Immune signaling is subsequently activated in neutrophils, macrophages and dendritic cells, resulting in pathogen removal. This elimination pathway is initiated by the binding of pathogen-derived molecules, called pathogen-associated molecular patterns (PAMPs), to PRRs expressed on phagocytes, leading to the formation of nascent phagosomes containing pathogens and their subsequent fusion to lysosomes. In particular, macrophages and dendritic cells are responsible for the antigen presentation of pathogen-derived molecules via major histocompatibility complex (MHC)-mediated pathways, resulting in the induction of acquired immune responses. TLRs are the best-characterized germline-encoded PRR proteins, and they transmit signals through several adaptor molecules [41]. TLR4 was the first member of the TLR family to be identified functionally [42]. TLR4 binds to lipopolysaccharide (LPS) with the support of GPI-anchored protein CD14, suggesting that TLR4 is activated at the sites of membrane microdomains [43]. The interaction of receptor molecules with membrane microdomains containing GM1 during LPS-induced cellular activation [44] was evaluated by fluorescence resonance energy transfer (FRET), which showed that TLR4 is associated with GM1-positive membrane microdomains after stimulation with LPS. A cholesterol-binding motif of TLR4 is regarded as critical for its translocation into membrane microdomains [45], suggesting that sphingolipids may not be involved in TLR4 signaling [46]. Recent atomistic molecular dynamics (MD) simulations regarding the TLR4 dimer complex showed that glucosylceramide (GlcCer) enhanced electrostatic interactions of the TLR4 extracellular domain with membranes [47]. This result suggests that the effects of GlcCer on LPS/TLR4 orientation affects LPS/TLR4 signaling through MyD88 adapter-like (Mal), also termed TIRAP. Thus, additional investigations are required to elucidate the mechanisms underlying the *cis*-interactions of TLR4 with membrane microdomain components. Cross-talk between neurotrophic receptors and TLR4 is also thought to be involved in neuroprotection mechanisms [48]. An extract from inflamed rabbit skin inoculated with vaccinia virus (Neurotropin^®^, NTP) was shown to control nerve growth factor (NGF) receptor TrkA-mediated TLR4-associated signaling through clusters of newly formed membrane microdomains in PC12 cells. GSL-enriched microdomains involve not only TLR4-mediated immune functions but also functions mediated by other members of the TLR family. For example, the binding of bacterial flagellin to asialoGM1 and TLR5 expressed on human lung epithelial cells was found to induce the autocrine release of ATP [49]. This released ATP binds to and activates ATP receptors in plasma membranes, leading to Ca^2+^ mobilization and Erk1/2 phosphorylation. GD1a on human monocytes binds to the subunit of type IIb *Escherichia coli* enterotoxin, promoting its interaction with the TLR2/TLR1 signaling complex and activating NF-κB [50].

## 4. GSL-Enriched Microdomains in Immune Functions

Several properties of neutrophils, including adhesion, migration and phagocytosis, are modulated by the CD11b/CD18 integrin, also called Mac-1, CR3 or α_M_β_2_ integrin [58]. Despite CD11b/CD18 cytoplasmic regions lacking catalytic activity, CD11b/CD18 can transduce signals inside the cells [59]. These results suggest that CD11b/CD18-mediated outside-in signaling requires partner molecules as a signaling platform. Microdomains enriched in the neutral GSL LacCer can not only bind microbial ligands but can act as a signaling platform. That is, LacCer-enriched microdomains are able to act as a PRR for pathogens. Indeed, LacCer was found to bind to various pathogens and their PAMPs, including *Candida albicans*-derived β-glucan (CSBG) and mycobacterial lipoarabinomannan (LAM) [20,60], and to form membrane microdomains coupled to various signaling molecules, including the Src family kinase Lyn, through their very long C24 fatty acid chains, transducing ligand-binding signals to the inside of the cells [18]. In addition, Lyn-coupled LacCer-enriched microdomains serve as a platform for CD11b/CD18-dependent outside-in and phagocytic signaling in human neutrophils [20,51,61]. LacCer-enriched microdomains are likely to interact in *cis* with the extracellular juxtamembrane region of CD18 [51]. In lung epithelial cells, the interaction of the *Pseudomonas aeruginosa*-derived virulence factor LecA with globotriaosylceramide (Gb3Cer) in the outer leaflet of plasma membranes was found to induce the formation of membrane domains enriched in saturated long fatty acyl chain-containing Gb3Cer species, the GPI-anchored protein CD59, phosphatidylinositol (3,4,5)-trisphosphate (PIP3) and flotillin, thereby promoting the efficient uptake of *P. aeruginosa* [52]. Mechanisms were suggested by which Gb3Cer and its associated molecules mediate signal transduction from extracellular to intracellular sites through transbilayer coupling. Thus, by providing a signaling platform, several types of GSL-enriched microdomains can regulate the function of receptors that lack catalytic moieties, such as CD11b/CD18. Moreover, the structures of GSLs, especially their fatty acid chains, may be a key component of GSL-enriched microdomains that act as signaling platforms.

GSLs may also be involved in MHC-mediated antigen presentation pathways. For example, MHC class II molecules have been reported to contain possible Gb3Cer binding sites [62]. Results suggested that Gb3Cer modulates MHC class II-mediated antigen presentation from B cells to helper T cells, although the molecular mechanisms of Gb3Cer-MHC class II binding are still unknown. Tumors, however, may limit MHC class I-mediated antigen presentation [63]. A recent study investigating the roles of GSLs and related enzymes in MHC class I pathways using genome-wide haploid genetic screening and CRISPR/Cas9 systems [53] found that, in the absence of signal peptide peptidase-like 3 (SPPL3) protease, high amounts of negatively charged neolacto-series GSLs interfere with the accessibility of MHC class I molecules for immune cell receptors, suppressing the activation of CD8^+^ T cells. In this pathway, SPPL3 catabolizes the glycosyltransferase B3GNT5, which generates neolacto-series GSLs, and controls the ability of MHC class I molecules to access their receptors. Thus, neolacto-series GSLs may affect antigen presentation and help tumor cells escape from immune surveillance [63]. In addition, the sialic acid residues on GSLs were found to be critical for MHC class I shielding [53]. The molecular basis of the interactions between GSL-enriched microdomains and antigen presentation-related molecules may provide critical information that can help in the treatment of immune disorders.

In addition to their roles in innate immune signaling, GSL-enriched microdomains are also essential in acquired immune signaling. During T-cell responses, lymphocyte function-associated antigen-1 (LFA-1) moves into membrane microdomains upon CD4 ligation, becoming associated mainly with GM3 [54]. At this time, phosphoinositide 3-kinase (PI3K) is mainly associated with GM1, and its association with p56lck was increased. During the reaction, LFA-1 becomes primarily associated with GM1. However, the mechanisms by which these gangliosides interact (directly or indirectly) with protein molecules remain unknown. a-Series gangliosides and asialo-series gangliosides have been implicated in the function and stimulation of T-cell receptors (TCRs) on CD4-positive (CD4^+^) and CD8-positive (CD8^+^) T cells, respectively [55], indicating the possibility that CD4 and CD8 interact with a-series and asialo-series gangliosides, respectively, through their common glycan structures [55,64]. Similarly, the ceramide structures of gangliosides may participate in these interactions. Although future studies are needed to address these possibilities, individual gangliosides may be involved in the movement of CD4 and CD8 to specific and correct locations in cell membranes [55,64].

Notch signaling is critical for T-cell development in the thymus [65]. Notch ligands, such as Dll1, may interact with GSLs through their GSL-binding motif [66], suggesting that the molecular interactions of protein receptors and ligands with GSLs may be associated with the regulation of T-cell functions. T cells play important roles in the pathogenesis of various autoimmune diseases, including systemic lupus erythematosus (SLE) [67]. GSL expression is dysregulated in CD4^+^ T cells from patients with SLE [68], and T cells from these patients show alternations in GSL recycling and turnover. Thus, GSL-enriched microdomains may be implicated in the pathogenesis of autoimmune diseases. GSL-enriched microdomains interact with B-cell receptors (BCRs) as well as participating in TCR-mediated functions. Indeed, GM1-enriched microdomains associated with BCR signaling may be critical for ganglioside-related B lymphocyte functions [56,69,70,71]. BCRs are indispensable for the B-cell clonal selection process and their differentiation into plasma cells. GM1-enriched microdomains may be involved in the compartmentalization of different types of BCRs, such as IgM and IgD types, expressed on mature B cells [56,70], although the mechanisms by which GM1-enriched microdomains modulate BCR signaling remain to be determined.

## 5. GSLs and Their Antibodies

Although GSL and its enriched membrane microdomains modulate a diverse array of immune responses through various signaling pathways, the pathogenesis of autoimmune diseases is thought to involve immune responses to GSL antigens. Healthy human sera contain a variety of anti-GSL antibodies, suggesting that endogenous GSLs act as antigens and induce B cells to produce antibodies [72,73]. However, infection by several pathogens, including *Campylobacter jejuni* and cytomegalovirus, leads to the production of autoantibodies against gangliosides. These findings suggest that autoantibodies play various pathogenic roles in autoimmune diseases, such as Guillain–Barré syndrome (GBS) [74]. The specificity of anti-GM1 antibodies in sera from patients with GBS varies, depending on clinical condition [75]. Interestingly, the specificities of the anti-LacCer monoclonal antibodies (mAbs) T5A7 and Huly-m13 differ from each other [76]. Whereas both T5A7 and Huly-m13 recognize LacCer on human neutrophils, T5A7 alone recognizes LacCer on mouse neutrophils. Furthermore, Huly-m13 is capable of precipitating LacCer antigens, whereas T5A7 is not [51]. T5A7 and Huly-m13 have distinct binding regions in LacCer-enriched microdomains [76], suggesting that the antigenic specificity of GSLs is complicated in plasma membranes. Thus, the specificities of mAbs against the same GSL antigens may reflect differences in the three-dimensional structural features of GSL-enriched microdomains. A recent study using antibodies against artificially synthesized GSLs showed that fatty acid chain length correlated with GSL antigenicity [77]. Moreover, the oligosaccharide structures in GSLs influence the class switching of induced antibodies [78]. Autoantibodies against LacCer are present in patients with encephalomyeloradiculoneuropathy (EMRN) [79], suggesting that antibody-mediated immune responses to GSL antigens may be implicated in the pathogenesis of several disorders, such as EMRN.

## 6. GSL-Enriched Microdomain-Mediated Apoptosis and Autophagy

Sphingolipid- and cholesterol-rich membrane microdomains serve as platforms for the recruitment and concentration of apoptotic signaling molecules at the plasma membrane [80,81,82,83]. There are two main apoptotic pathways: the extrinsic or death receptor pathway and the intrinsic or mitochondrial pathway. During the induction of apoptosis, Fas/CD95, Fas-associated death domain (FADD)-containing protein and procaspase-8/10 (all the DISC components) are recruited into lipid microdomains. Once multimeric complexes (DISC) are formed, the number of procaspase-8 molecules increases, resulting in the self-activation of procaspase-8. This results in the activation of a caspase cascade and the initiation of apoptosis [84]. The formation of clusters of apoptotic signaling molecule-enriched rafts (CASMERs) could be a major regulator of apoptotic signaling [84,85]. The recruitment of Bid (BH3-interacting domain death agonist) to CASMERs facilitates interactions between death receptor-related extrinsic apoptosis signaling and mitochondrial-related intrinsic apoptosis signaling, thereby amplifying apoptosis. CASMER formation and the compositions of CASMERs are dependent on cell types and stimulants [85,86]. Cholesterol is a critical constituent of lipid microdomains, with displacement of cholesterol disrupting CASMER formation [80,84]. Cancer cells contain high levels of cholesterol and cholesterol-rich microdomains, making them prone to form CASMERs. Novel CASMERs can promote apoptotic machinery of tumor cells, with CASMER formation making cancer cells more vulnerable. Thus, enhancing CASMER formation may be a potential therapeutic target in cancers [84].

GSLs play pivotal roles in apoptotic pathways. GSLs form lipid microdomains in the membranes of subcellular compartments, as well as in plasma membranes. These subcellular GSL-enriched lipid microdomains mediate important signaling pathways involved in various physiological functions. GM3 and disialoganglioside GD3 are abundant components in the lipid microdomains of human lymphocytic cells [87,88]. The treatment of human lymphoblastoid T cells with anti-CD95/Fas triggers an association of GM3 and caspase-8, indicating that gangliosides are structural components of the membrane multi-molecular signaling complex involved in the apoptosis pathway mediated by the CD95/Fas receptor [57]. GD3 is a minor ganglioside in most normal tissues but is highly expressed in a variety of tumors, resulting in its original description as a tumor-associated ganglioside [89]. GD3 interacts directly with isolated mitochondria and induces organelle swelling and the production of reactive oxygen species (ROS), cytochrome C, apoptosis-inducing factor (AIF) and caspase-9 [89]. Both intracellular and exogenously added GD3 were able to interact with mitochondria. GD3 induces mitochondrial permeability transition, occurring prior to apoptosis, in MH1C1 cells [90]. The opening of the permeability transition pore complex (PTPC) in mitochondria is controlled by bcl-2, which blocks apoptosis induced by both endogenous and exogenous GD3 [91]. TNF-α stimulates a physical association with GD3 in the mitochondria of rat hepatocytes [92]. Moreover, GD3 interacts with voltage-dependent anion channels (VDAC-1) on mitochondrial membranes. As a structural component of multi-molecular complexes that include VDAC-1, Bcl2-family proteins (Bax and t-Bid) and fission proteins, GD3 is involved in the opening of mitochondrial permeability transition pores [93]. In melanocyte, the expression of the GD3 synthase gene is oppositely regulated by TNF-α and cAMP [94]. Taken together, these findings indicate that GSLs play pivotal roles in apoptotic pathways.

In innate immunity, apoptosis is a self-destructive response, by which tissue-resident macrophages eliminate neutrophils from inflamed tissues. These processes are crucial for limiting injury to inflammatory tissues and the subsequent resolution of inflammation [95]. Similar to LacCer, the unique glycophospholipid, phosphatidylglucoside (PtdGlc), is highly expressed on the outer leaflet of the plasma membrane of human neutrophils [96]. However, PtdGlc and LacCer form distinct microdomains on the cells and mediate distinct signaling pathways [95]. PtdGlc has also been reported to be a marker of neutrophil differentiation [97,98]. Importantly, the cross-linking of PtdGlc with the anti-PtdGlc antibody induces an association between PtdGlc-enriched microdomains and Fas [95], and the anti-PtdGlc antibody induces apoptosis of human neutrophils. Thus, microdomain-mediated apoptosis is thought to be an essential process in the innate and adaptive immune systems.

Although autophagy and apoptosis represent distinct cellular processes with fundamentally different biochemical and morphological features, they are highly connected to each other [83,99,100]. Autophagy is triggered in response to many stresses that ultimately lead to apoptosis. In the presence of persistent stress, autophagy is unable to support cell survival, resulting in the activation of apoptosis to ensure the effective elimination of these cells without causing local inflammation [99]. Thus, apoptosis and autophagy can cooperate, antagonize or assist each other, differentially influencing the fate of cells [83]. Three distinct forms of autophagy have been identified: macroautophagy, microautophagy and chaperone-mediated autophagy [101,102]. In macroautophagy, the cell forms a double-membrane sequestering compartment, called the phagophore. Phagophore formation is initiated by the ULK1 complex, the class III PtdIns 3K complex (PIK3C3) and autophagy-related (ATG) genes (ATG5–ATG12–ATG16L complex). The expanding membrane of the phagophore closes around the cargo and separates it from the endoplasmic reticulum (ER) to form a mature autophagosome. These mature autophagosomes fuse with lysosomes, forming autophagolysosomes. Their contents are degraded by proteases, and the resulting macromolecules are transported into the cytosol for recycling [102,103]. GD3 is associated with phosphatidylinositol (3) phosphate (PtdIns3P) and microtubule-associated protein light chain 3 (LC-3) in fibroblasts, indicating the importance of GD3 in the timing of the initiation phase of autophagy and in autophagosome biogenesis [104]. GD3 is also associated with LAMP-1 at later time points, suggesting that GD3 also plays a role in the maturation of autophagosomes into autolysosomes [104]. Autophagosomes form at ER–mitochondria contact sites [105]. GD3 interacts with lipid microdomains in mitochondria-associated membranes (MAMs) and contributes to the assembly of autophagosome [106]. Thus, lipid microdomains in MAMs may play pivotal roles in the scrambling organelle that leads to the formation of autophagosomes. Autophagosomes may also assemble at ER–plasma membrane contact sites [107,108]. Extended synaptotagmins (E-Syts) are proteins that act as key regulators of ER–plasma membrane tethering and are involved in autophagosome biogenesis. Vacuole membrane protein 1 (VMP1), which is enriched in ER microdomains, promotes the association of the class III PtdIns 3-kinase complex (PIK3C3) with E-Syt-containing domains, enhancing PtdIns3P synthesis [107,108]. These findings suggest that contacts between the ER and organelles are indispensable in autophagy and organelle biogenesis. ER-driven contacts with, for example, the plasma and mitochondrial membranes, may act as local platforms for PIK3C3. MAMs are expected to be important targets for the treatment of autophagy-related diseases. The determination of the involvement of GSLs with the formation of a platform containing autophagy-related proteins is essential in the development of novel treatments for many human diseases, such as cancer and neurodegenerative diseases, including Parkinson’s disease, Alzheimer’s disease and amyotrophic lateral sclerosis [109].

## 7. GSLs as Immunomodulators

As mentioned above, GSL-enriched microdomains transduce signals into cells through receptors and signaling molecules, mediating a number of physiological functions. Exogenously added GSLs alter receptor/signaling molecule-mediated cellular signaling (Table 2). Therefore, understanding the mechanisms by which exogenous GSLs modulate immunological functions is linked to the development of therapeutic agents for a number of diseases. To date, the roles of exogenous gangliosides in the immunological functions mediated by TLRs have been investigated. The preincubation of monocytes or immature dendritic cells (DCs) derived from peripheral blood mononuclear cells (PBMCs) with GM1, GD1a and GD1b was found to inhibit the production of cytokines, such as IL-6, IL-12 and TNF-α, through a broad range of TLRs, including TLR2, TLR3, TLR4, TLR6 and TLR7/8 [110]. In addition, gangliosides were found to upregulate the expression of the TLR signaling inhibitor, IL-1 receptor associated kinase-M (IRAK-M), without inducing cytokine production. GD1a and GM1 were shown to reduce the LPS-induced biological effects in PC12 and epithelial cells, respectively [111], and to prevent the LPS-induced translocation of TLR4 into membrane microdomains, suggesting that GD1a and GM1 protect cells against LPS. The pretreatment of a mouse macrophage-like cell line, Raw 264.7, with sulfated galactocerebroside (sulfatide, SM4) was found to hinder LPS-induced TLR4 colocalization with CTxB-positive ganglioside-rich microdomains, suppressing the secretion of a sepsis mediator, high mobility group box 1 (HMGB1) [112]. GSLs are also thought to modulate receptor-mediated signaling by binding to these receptors (Table 2). For example, globotetraosylceramide (Gb4Cer) from vascular endothelial cells inhibited LPS binding to TLR4 and attenuated TLR4-MD-2-mediated LPS signaling [113]. In contrast, the treatment of mouse bone marrow-derived macrophages (BMDMs) and human monocytes with Gb3Cer/Gb4Cer was found to enhance TLR4-mediated inflammation, suggesting that the elevation of Gb3Cer/Gb4Cer by consumption of a high-fat diet positively regulates TLR4-mediated inflammatory responses [114]. Further studies are required to better understand the mechanisms underlying the binding of Gb4Cer to the TLR4-MD-2 complex. In addition, differences in molecular complex formation and receptor expression levels among cell types might affect GSL-receptor binding and signaling. Recently, the effect of GM3 on TLR4 signaling was reported to depend on the fatty acid structure of GM3 [115]. Interestingly, GM3 species containing saturated very long-chain fatty acids (C22:0, C24:0, hC24:0) enhanced LPS/HMGB1-associated TLR4 signaling in monocytes, whereas GM3 containing long-chain fatty acids (C16:0, C18:0) or unsaturated very long- chain fatty acids (C24:1) inhibited this activation. More recently, it was reported that C12 or C16-SM4 activates TLR4-MD-2 in mouse macrophages, whereas those GSLs antagonize TLR4-MD-2 activation in a human macrophage-like cell line (PMA-differentiated THP-1) [116]. β-GlcCer was shown to act as an endogenous ligand for macrophage inducible C-type lectin (Mincle) and an immunostimulatory factor in response to cell damage [117]. A further understanding of the mechanisms by which different molecular species of GSLs modulate immune receptors as agonists/antagonists may suggest new therapeutic strategies for several disorders.

Exogenous LacCer has been found to be involved in cell adhesion, angiogenesis, the generation of ROS and inflammation [123,124]. It has been reported that, in human neutrophils and monocytes, exogenously added LacCer upregulates the expression of CD11b/CD18 and promotes their adhesion to the endothelium and subsequent entry into the endothelium, inducing inflammation and atherosclerosis [123,125]. Moreover, exogenously added LacCer activates cytosolic phospholipase A2 (cPLA2) to generate arachidonic acid, a precursor to prostaglandins that mediate inflammation [123,125]. The human acute myeloid leukemia cell line HL-60 can be differentiated into cells of the neutrophil lineage by incubation with dimethyl sulfoxide (DMSO). These D-HL-60 cells, however, are unable to exert LacCer-mediated innate immune responses [25,51], because their plasma membranes contain few very long fatty acid-containing LacCers (C24-LacCer) [18]. Importantly, exogenously added C24- but not C16-LacCer directly interacts with Lyn through the binding of the C24 fatty acid chains to the palmitic chains of Lyn [61]. This resulted in the reconstruction of LacCer-enriched microdomains, which are coupled to several signaling molecules. These reconstructed C24-LacCer-containing microdomains can act as signaling platforms for several neutrophil functions, including chemotaxis, phagocytosis and superoxide generation. In contrast to the previously known properties of β-glucans [126], β-glucan binding to C24-LacCer-containing microdomains was found to trigger the Lyn-mediated phosphorylation of phosphatase SHP-1 and to reduce FcγRIIA affinity in dimethylformamide (DMF)-treated HL-60 cells [118]. These findings suggest that β-glucan binding to C24-LacCer leads to inside-out signals that reduce FcγRIIA affinity. LacCer can bind to a variety of PAMPs, including β-glucan [64]. Exogenous C24-LacCer enhanced the phagocytosis not only of β-glucan-rich zymosan particles but also of mycobacteria [20]. Therefore, exogenous LacCer may alter the microenvironment of microdomains and positively or negatively regulate microdomain-associated immune receptor signaling.

CD1d presents GSLs as antigens to cells of the innate immune system, such as natural killer T (NKT) cells. These cells are involved in the regulation of innate and adaptive immune responses against cancers, infectious diseases and inflammatory diseases [127]. A marine sponge-derived α-GalCer was shown to be a lipid antigen molecule that activates NKT cells [128] and enhances immune responses to various infectious microorganisms and cancers [129]. α-Linked glycosylceramides are identified as endogenous ligands on NKT cells [130]. These findings suggest that, as a modulator of NKT cells, GSLs can be therapeutic in patients with several types of immune disorders.

The pretreatment of murine bone marrow-derived DCs (BMDCs) with gangliosides was found to facilitate the development of regulatory T-cell activity [119]. Tumor-derived gangliosides suppress lytic function in CD8^+^ cytotoxic T lymphocytes (CTLs) by preventing the TCR-induced release of lytic granules [120]. Moreover, gangliosides cooperate with interferon (IFN)-γ to inhibit the immunostimulatory activity of DCs in an inflammatory environment [121]. In contrast, the ganglioside GQ1b facilitates cytokine production by T cells, without altering cytokine production by B cells and monocytes [122]. The co-culture of B cells with GQ1b-treated T cells enabled B cells to produce immunoglobulin (Ig). GQ1b may indirectly enhance the B-cell production of Ig by inducing the T-cell production of IL-6 and IL-10. Thus, extracellular or exogenously added gangliosides are likely to regulate adaptive immune responses.

## 8. GSL-Enriched Microdomains as Entry Sites for Pathogens and Toxins

As mentioned above, GSL-enriched microdomains serve as signaling platforms for a variety of physiological functions through their *cis*- or *trans*-interactions with protein receptors. GSL-enriched microdomains provide the entry sites for foreign materials by binding directly to pathogens and toxins. To date, a large number of pathogens and toxins have been reported to gain access to host cells through their *trans*-interaction with GSLs. For example, polyomavirus must bind to GD1a and GT1b to enter human erythrocytes [131]. In addition, the N-terminal domain (NTD) of the SARS-coronavirus 2 (SARS-CoV-2) spike protein has a ganglioside-binding domain [132,133], and angiotensin-converting enzyme-2 (ACE2), a major receptor for SARS-CoV-2, colocalizes with GM1 [134]. Because the mechanisms by which GM1-enriched microdomains are associated with ACE2 remain unclear, the determination of these molecular mechanisms may be critical in the development of treatments for COVID-19. Shiga toxin (Stx) and its verotoxin B subunit have been found to bind preferably to Gb3Cer expressed on human epithelial and endothelial cells [135,136,137,138]. Based on current knowledge, Stx subtypes Stx1a and Stx2a are known to prevalently bind to Gb3Cer from primary human brain microvascular endothelial cells (pHBMECs) and primary human renal glomerular endothelial cells (pHRGECs), respectively, although both Stx1a and Stx2a show significant lower binding to Gb4Cer [139,140]. Gb3Cer predominantly distributes in the DRMs of both pHBMECs and pHRGECs [139,140]. Such preferential distribution of Gb3Cer to DRMs suggests an association of Gb3Cer with membrane microdomains and appearance of the major Stx receptor in a microdomain environment [140]. In addition, the prevalent lipoforms of Gb3Cer and Gb4Cer in pHBMECs and pHRGECs were those with Cer (d18:1, C16:0), Cer (d18:1, C22:0) and Cer (d18:1, C24:1/C24:0) [140]. More recently, Gb3Cer and Gb4Cer with Cer (d18:1, C16:0), Cer (d18:1, C22:0) and Cer (d18:1, C24:1/C24:0) were identified as the dominant lipoforms of primary human renal cortical epithelial cells (pHRCEpiCs) [141]. Gb3Cer and Gb4Cer lipoforms carrying a C24:1 or C24:0 fatty acid chain could be involved in the interdigitation between the fatty acyl chains of the two leaflets of the plasma membrane microdomains [141]. Stxs are multifunctional proteins capable of activating multiple stress signaling pathways, resulting in apoptosis, autophagy or induction of innate immune responses [142,143]. Therefore, long acyl chain-containing Gb3Cer/Gb4Cer in the plasma membrane microdomains may play critical roles in multiple Stxs-mediated immune responses. Although Gb4Cer is not involved in the binding or internalization of parvovirus B19 (B19V), Gb4Cer is important in a post-internalization step prior to the delivery of viral DNA into the nucleus [144]. *Streptococcus suis* SadP adhesin from systemic subtype P_N_ strains binds to Gb4Cer through its amino acid asparagine 285 [145]. Asialo GM1 (GA1) expressed by epithelial cells binds to *Pseudomonas aeruginosa*, *Bifidobacterium bifidum* and *Lactobacillus* [146,147], and GM1 was found to bind to simian virus 40 (SV40) [131] and *Brucella suis* [148]. This ganglioside can bind to CTxB [149,150], and GM1 expressed on epithelial cells binds to *Escherichia coli* enterotoxin [151]. GM1 containing long acyl chains is essential for the membrane invaginations of SV40 [152]. However, GM1 species containing unsaturated or short acyl chains is reported to be critical for the GM1-mediated transcytosis of CTxB [153]. The recent study by Kabbania demonstrated that the clustering of GM1 is required for CTxB-induced membrane curvature in model membranes, but the ceramide structure is not likely to play a determinant role in this mechanism [154]. Therefore, pathogen/toxin-induced GM1-mediated physiological functions may be altered by the structural differences of the GM1 ceramide portion. In addition to GM1, other gangliosides, particularly fucosylated GM1 (Fuc-GM1), are involved in binding to CTB [155,156]. Galactosylceramide (GalCer) binds to the influenza A virus (IAV) [157] and the norovirus GII.4 [158]. In addition to GalCer, Gb3Cer and GM3 were shown to act as entry receptors of human immunodeficiency virus (HIV-1) [159], suggesting that GSL-enriched microdomains are involved in stabilizing HIV-1 attachment to the cell surface and in the promotion of co-receptor recruitment [160,161,162]. Intracellular parasites, such as *Mycobacterium tuberculosis*, enter into host phagocytes via membrane microdomains, which enable them to survive inside cells [163]. These findings suggest that, to promote their survival, intracellular parasites exploit GSL-enriched microdomains to manipulate host microbicidal pathways. LacCer, which is expressed on intestinal epithelial cells as well as on neutrophils, binds to various microorganisms, such as *Mycobacterium avium–intracellulare* complex (MAC), *M. tuberculosis*, *Candida albicans*, *Bacillus dysenteriae*, *Bordetella pertussis*, *E. coli* and *Propionibacterium freudenreichii* [64]. In addition, the Gram-negative bacterium *Edwardsiella tarda*, a causative agent of economic damage in aquaculture, can bind LacCer but not GlcCer [164]. LacCer is a major GSL component in human and bovine milk [165], indicating that milk-derived LacCer may block pathogen binding in the intestines, protecting the host from invading pathogens. The finding that several species of microorganisms bind to LacCer suggests the presence of common structural patterns on the membranes of these microorganisms that can be recognized by LacCer-enriched microdomains. Indeed, LacCer-enriched microdomains recognize PAMPs differentially expressed by fungi and mycobacteria [20,60,64]. The pathogenic fungus-derived β-glucan, CSBG, is composed of a β-1,3 glucopyranose glucan backbone, with β-1,6 long glucopyranose side chains and β-1,3 monoglucopyranose branches [166]. GSLs with a terminal galactose residue, such as GalCer, LacCer and Gb3Cer, are essential for binding to CSBG [60]. Similarly, these types of GSLs bind to yeast-derived PGG-glucan [167], which has a structure similar to that of CSBG [168]. In contrast, the glycolipid lipoarabinomannan (LAM), which is abundantly expressed on the cell walls of mycobacteria [169], contains a mannan core structure, consisting of a linear backbone of α-1,6 mannopyranose with α-1,2 mannopyranose side branches [170]. Although this structure is a common motif among mycobacterial species [170], the LAM of pathogenic mycobacteria, including *M. tuberculosis*, has a terminal mannose cap, ManLAM, whereas the LAM of non-pathogenic species has a terminal phospho-myoinositol cap (PILAM) or no cap LAM [171]. LacCer binds to both ManLAM and PILAM [20], suggesting that LacCer-enriched microdomains recognize the common α-1,2 monomannose side branching mannan core of LAM present in mycobacterial species. GSL-enriched microdomains may recognize the common three-dimensional structures among different PAMPs through carbohydrate–carbohydrate interactions. The sugar moieties of gangliosides interact with the polysaccharide moieties of *Shigella* LPS [172], thereby facilitating the binding of bacteria to human CD4^+^ T cells. These findings suggest that specific carbohydrate–carbohydrate interactions between the sugar moieties of GSLs and PAMPs may be implicated in various infectious diseases.

## 9. Intracellular Interactions between GSL-Enriched Microdomains and Pathogens

GSL-enriched microdomains in plasma membranes function as entry sites for pathogens and toxins. The uptake of pathogens into cells leads to nascent phagosome formation, resulting in the fusion of lysosomes to pathogen-containing phagosomes (phagosome maturation). However, the molecular mechanisms by which GSL-enriched microdomains interact intracellularly with pathogens, such as during the phagosome maturation process, remain to be elucidated. Recently, genome-wide CRISPR screening showed that the enzymes ceramide synthase, fatty acid elongase and sphingomyelin synthase 1 (SMS1) are involved in phagocytosis [173]. In addition, sphingomyelin biosynthesis was reported to be critical for the uptake of *M. tuberculosis* by human macrophages [174]. Quantitative lipidomic analysis demonstrated that ceramide synthase 2 (CerS2) is enriched in early phagosomes and that the amounts of C24 ceramides are increased in late phagosomes [175]. LacCer-enriched microdomains in human neutrophils are essential not only in the uptake of bacteria but also in the maturation process of bacteria-containing phagosomes [20,25,51]. Interestingly, the pathogenic mycobacteria, *M. tuberculosis* and MAC, were found to inhibit the association of LacCer-enriched microdomains with the signaling molecule Hck, leading to phagosome maturation arrest. In addition, SM is distributed as clusters in the inner leaflet of plasma membranes of human neutrophils [2], suggesting the involvement of SM in intracellular events. More recently, SM was reported to be exposed on the cytosolic side of damaged phagosomal membranes around *Salmonella* or *Listeria*, with galectin-8 localizing to the phagosomal membranes [176,177]. The cytosolic exposure of SM by Ca^2+^-activated scramblase was recently reported to mediate lysosomal repair independently of the endosomal sorting complex required for the transport complex (ESCRT) [178]. Although the mechanisms by which pathogenic mycobacteria survive inside host cells remain unclear [179], an exploration of the involvement of cross-talk between GSL-enriched microdomains and other sphingolipids in intracellular events, such as phagosome maturation, may be key to the development of new treatments for mycobacterial infections.

Some viruses exploit GSLs to enter into and survive inside host cells. Indeed, GM3 was reported to be essential for the replication of dengue virus (DENV) [180]. SM, Cer and GlcCer are enriched in Zika virus (ZIKV) particles [181]. Patients who are infected with ZIKV and develop the neurological autoimmune disorder Guillain–Barré syndrome (GBS) [182] have high levels of antibodies to GSLs: GM1, GM2, GA1, GD1a, GD1b and various other gangliosides [183,184]. Although GSLs are required for the replication of ZIKV, they are not necessary for virus entry into host cells. ZIKV alters host lipid composition, modulating sphingolipid pathways [185], and may therefore manipulate ceramide-related metabolism. Mutations in SARS-CoV-2, a new type of coronavirus, result in alterations in the spike protein recognized by host ACE-2 receptors, allowing the virus to escape from host recognition and elimination systems [186]. SARS-CoV-2 may bind to the sialic acid moieties of the gangliosides expressed on host cell surfaces [134]. The concentrations of circulating sphingosine-1 phosphate (S1P) were found to be lower in patients with more severe clinical scores [187,188]. Because S1P is associated with the induction of inflammatory responses, the S1P analogue FTY720 (fingolimod), which acts as a S1P receptor antagonist, may suppress hyper-inflammation, although the immunosuppressive effects of FTY720 alone are not negligible. Together, GSL-enriched microdomains and sphingolipid-derived metabolites may be essential for infection by emerging viruses, such as SARS-CoV-2, and may therefore be a target for the treatment of viral infections.

The infection of mouse alveolar macrophages with *Pseudomonas aeruginosa* results in the activation of acid sphingomyelinase (aSMase) and the formation of ceramide-enriched microdomains [189]. These ceramide-enriched microdomains are required for the *P. aeruginosa*-induced activation of nicotinamide adenine dinucleotide phosphate (NADPH) oxidase, which produces reactive oxygen species (ROS). ASMase activation leads to the internalization of *P. aeruginosa* and the induction of apoptosis. *P. aeruginosa*-derived phospholipase C and alkaline ceramidase are involved in sphingolipid metabolism [190,191,192]. *Chlamydia psittaci* is able to obtain sphingolipids from host cells, thereby surviving in host cells [193,194], whereas *C. trachomatis* recruits CERT, SMS1, SMS2 and VAP-A to inclusion bodies, acquiring ceramide and metabolizing it to SM [195]. *C. trachomatis* is able to proliferate in SMS1/SMS2 double-knockout HeLa cells but not in SMS1/SMS2/CERT triple-knockout cells [196]. *C. trachomatis* can exploit the signaling pathway involving Akt and its 160 kDa substrate AS160 to increase the delivery of sphingolipids to inclusion bodies through Rab14 [197]. These reports suggest that several intracellular pathogens, such as *Pseudomonas* and *Chlamydia*, control sphingolipid metabolism pathways and vesicular transport.

Gram-negative pathogenic bacteria are known to use the type III secretion system (T3SS) to induce pore formation on host membranes [198,199]. *Salmonella* uses T3SS to rupture phagosomal membranes [176,177], releasing internalized bacteria into the cytoplasmic region of host cells and inducing autophagy (xenophagy) to eliminate these bacteria [200,201]. Recently, LC3-associated phagosomes (LAPsomes) were shown to be a novel form of non-canonical autophagy [202,203]. LAPsomes may function as regulators of the immune system. Indeed, LAPsome-deficient macrophages show reduced efferocytosis activity, and mice with LAPsome-deficient macrophages develop a lupus-like autoimmune disorder [204]. In addition to efferocytosis, microbicidal activity is reduced in LAPsome-deficient macrophages [205]. However, there is less evidence regarding the relationships between sphingolipids and LAPsomes. Some proteins in *Legionella pneumophila* are similar to eukaryotic sphingolipid metabolic enzymes [206,207,208]. In particular, *L. pneumophila* impairs autophagy by preventing host sphingosine metabolism through an S1P lyase, *LpSpl*. *Salmonella* suppresses Akt/mTOR signaling-mediated autophagy responses by recruiting focal adhesion kinase (FAK) to *Salmonella*-containing vacuoles (SCV) [209]. Together, these findings indicate that intracellular pathogens use GSL-enriched microdomains and sphingolipid metabolism to survive inside host cells. To eliminate internalized pathogens, GSL-enriched microdomain-associated signaling and sphingolipid metabolism pathways may be essential for vesicular transport and autophagy. However, the mechanisms involving the interplay between these two pathways remain to be determined.

## 10. Perspective

A huge number of investigations have been carried out to elucidate the roles of GSL-enriched microdomains in a wide range of physiological functions. In particular, research advances in the field of immunology provide insights into the mechanisms of how GSL-enriched microdomains modulate immune functions through multiple pathways. At present, investigators have understood the molecular mechanisms by which specialized GSL-enriched microdomains directly interact in *cis* with various protein receptors and mediate immune signaling. A better understanding of the mechanism of interaction between GSL-enriched microdomains and protein receptors in different aspects of pathogenesis may provide novel therapeutic targets for many types of diseases. GSL-enriched microdomains directly interact in *trans* with pathogens and their toxins and provide pathogens with entry sites into host cells. Therefore, a further characterization of the binding specificities of GSLs to pathogen-associated molecules would lead to the design and development of new drugs that prevent infection. Similarly, the characterization of anti-GSL antibodies may bring a significant benefit to the elucidation of the pathogenic mechanism of autoimmune diseases. In recent years, advanced technologies, such as genome-wide screening using CRISPR/Cas9 systems, have been applied to the studies of GSL functions [210,211,212]. The lipidomes and transcriptomes of individual human cells by coupling high-resolution mass spectrometry imaging to single-cell transcriptomics may also open a new paradigm for unravelling unidentified functions of GSLs [213]. These advanced technologies, along with anti-GSL antibodies, may help to better understand the functions of GSL-enriched microdomains and may lead to the development of new pharmacological drugs for the treatment of various immune disorders.

## Figures and Tables

**Table 1:** GSLs and receptor-mediated immune signaling.

GSLs	Co-Receptors	Cell Type	Immune Signaling	Ref. No.
GlcCer	TLR4	Macrophages	Impact on LPS/TLR4 orientation and Mal-associated signaling	[47]
GA1	TLR5	Lung epithelial cells NCIH292	Flagellin-mediated autocrine release of ATP	[49]
GD1a	TLR2/TLR1	Monocytes	LT-IIb-B_5_-mediated NFκB activation	[50]
LacCer	CD11b/CD18	Neutrophils	Lyn and Akt activations, and the resulting phagocytosis of zymosan and mycobacteria	[20,51]
Gb3Cer	CD59	Lung epithelial cells H1299	PIP3 and flotillin-associated uptake of *P. aeruginosa*	[52]
Neolacto-series GSLs	MHC class I	HAP1 cells	Interference of the accessibility of MHC class I molecules for immune cell receptors and the resulting suppression of CD8^+^ T-cell activation	[53]
GM1, GM3	CD4, LFA-1	T-cell line	PI3K and p56lck-associated T-cell responses	[54]
a-Series gangliosides	CD4, TCR	T cells	Helper T-cell activation	[55]
Asialo-series gangliosides	CD8, TCR	T cells	Killer T-cell activation	[55]
GM1	IgM-BCR	Immature B cells	Removal of autoreactive immature B cells (apoptosis)	[56]
GM3	CD95/Fas	T cells	Formation of death-inducing signaling complex upon CD95/Fas engagement (apoptosis)	[57]

**Table 2:** GSLs as immune regulators.

GSLs	Immune Functions	Ref. No.
GM1, GD1a, GD1b	Inhibition of TLRs (TLR2, 3, 4, 6 and 7/8)-mediated IL-6, IL-12 and TNF-α production in monocytes and immature DCs	[110]
GM1, GD1a	Inhibition of LPS-induced biological effects in PC12 and epithelial cells	[111]
SM4	Inhibition of LPS-induced TLR4 colocalization with CTxB-positive ganglioside-rich microdomains and HMGB1 secretion in Raw 264.7 cells	[112]
Gb4Cer	Inhibition of LPS binding to TLR4 and attenuation of TLR4-MD-2-mediated LPS signaling in vascular endothelial cells	[113]
Gb3Cer/Gb4Cer	Enhancement of TLR4-mediated inflammation in mouse BMDMs and human monocytes	[114]
GM3 (C22:0, C24:0 or hC24:0 fatty acid)	Enhancement of LPS/HMGB1-associated TLR4 signaling in monocytes	[115]
GM3 (C16:0, C18:0 or C24:1 fatty acid)	Inhibition of LPS/HMGB1-associated TLR4 signaling in monocytes	[115]
SM4 (C12 or C16 fatty acid)	Activation of TLR4-MD-2 in mouse macrophages	[116]
SM4 (C12 or C16 fatty acid)	Antagonizing effect on TLR4-MD-2 activation in human macrophage-like PMA-differentiated THP-1 cells	[116]
β-GlcCer	Immunostimulatory factor upon cell damage, endogenous ligand for Mincle	[117]
LacCer (C24:0 or C24:1 fatty acid)	Enhancement of activated Lyn-mediated neutrophil functions (chemotaxis, phagocytosis and superoxide generation) in DMSO-treated HL-60 cells	[18,20,51,61]
LacCer (C24:0 or C24:1 fatty acid)	Induction of β-glucan binding-dependent SHP-1 phosphorylation through Lyn and the resulting reduction of FcγRIIA affinity in DMF-treated HL-60 cells	[118]
Gangliosides	Facilitation of the development of regulatory T-cell activity in murine BMDCs	[119]
Gangliosides (tumor derived)	Inhibition of lytic function in CD8^+^ CTLs	[120]
Gangliosides	Cooperative role with IFN-γ to inhibit the immnostimulatory activity of DCs	[121]
GQ1b	Facilitation of T-cell-mediated cytokine production, which possibly involves indirect enhancement of B-cell production of Ig	[122]
